
JOURNAL INFORMATION
==============================
NLM Title Abbreviation: Aesthet Surg J Open Forum
Journal ID: Aesthet Surg J Open Forum
NLM Journal ID: 101771713
Journal ID: asjopenforum

Aesthetic Surgery Journal. Open Forum

EISSN: 2631-4797

ARTICLE INFORMATION
==============================
PMCID: PMC7197617
PMID: 33786425
DOI: 10.1093/asjof/ojaa018
Article ID: ojaa018
Article version: 1
Subjects: Editorial Commentary, AcademicSubjects/MED00987

Six Feet Apart: Lessons Learned from COVID-19 and Social Distancing

http://orcid.org/0000-0002-6217-2604 Cress Phaedra BA
Corresponding Author: Ms Phaedra Cress, 11262 Monarch St., Garden Grove, CA 92841, USA.Email: phaedra@surgery.org; Twitter: @phaedracressExecutive Editor of Aesthetic Surgery Journal and ASJ Open Forum.

Electronic publication date: 2020 Apr 29
Electronic Location ID: ojaa018
Received 2020 Apr 5
Copyright: © 2020 The American Society for Aesthetic Plastic Surgery, Inc.
Copyright year: 2020
License: This is an Open Access article distributed under the terms of the Creative Commons Attribution License (http://creativecommons.org/licenses/by/4.0/), which permits unrestricted reuse, distribution, and reproduction in any medium, provided the original work is properly cited.
License URL: https://creativecommons.org/licenses/by/4.0/

article-lifecycle: PAP
edited-state: accepted-manuscript

==============================

**Six Feet Apart: Lessons Learned from COVID-19 and Social Distancing**

Phaedra Cress, BA

Ms Cress is the Executive Editor of *Aesthetic Surgery Journal* and *ASJ Open Forum*.

Corresponding Author: Ms Phaedra Cress, 11262 Monarch St., Garden Grove, CA 92841, USA.
Email: phaedra@surgery.org; Twitter: @phaedracress

Disclosures: Ms. Cress serves as Executive Editor of *Aesthetic Surgery Journal Open Forum.*

Funding: The author received no financial support for the research, authorship, and publication of this article.

Accepted Manuscript

© 2020 The American Society for Aesthetic Plastic Surgery, Inc.
This is an Open Access article distributed under the terms of the Creative Commons Attribution License (http://creativecommons.org/licenses/by/4.0/), which permits unrestricted reuse, distribution, and reproduction in any medium, provided the original work is properly cited.

Today is day 50 of my home quarantine. It is April 23, 2020.

The Coronavirus (or more technically COVID-19, CO for corona, VI for Virus and D for disease and 19 because of an early iteration's nomenclature—2019 novel coronavirus)—is an upper respiratory tract illness that has changed the world as we know it. We have begun ticking off the number of days in isolation from friends, family, and co-workers as a testament to who's been at it the longest and to remember how much longer we'll be at it until the curve is flattened and it's back to business as usual.

To avoid contracting the virus, we have been advised by the Centers for Disease Control (CDC) to remain six feet apart from other people. This is called social distancing, a new term introduced into our vocabulary and, some say, our new normal.

Though I am not a plastic surgeon like many of you reading this, I represent the "other half" whom you may be sheltering in place with (assuming you're not married to an essential worker). Until recently, the supply chain, and how all those glorious Amazon Prime boxes get to my house, was not something I gave any thought to. Supply-chain problems were not on the nightly news. Some of you were early adopters and others are still catching up. But what we know is that all our lives have received an unsolicited hard re-boot.

We're all feeling the enormity of the situation from learning to work from home (with spouses and children we now home school in tow), to avoiding physical contact at all costs, to finding food and other essentials we took for granted in the past, and learning how to craft our own masks, since the N-95 masks (3M, St. Paul, MN) are all backordered or being used by medical professionals and essential workers.

But there is a silver lining. New opportunities for innovation are happening all around us every day. New relationships are being formed. Old ones are being renewed. Communities are showing gratitude, every night, to the essential healthcare workers risking their own lives on the front lines. Whether it's singing or bells ringing, clapping, or pots banging, at 7pm Eastern every night, we let them know we appreciate their sacrifice. The Aesthetic Society has asked those of us who are staff: what can we do to help our members? They quickly began to triage and innovate to serve members immediately.

Today I asked myself these questions:

Accepted Manuscript

**What Have I Accomplished in the Past Month of Being Quarantined At Home?**

I started reading a book on reflexology, I made a video with my sons, hosted 2 birthdays on Zoom (Zoom Video Communications, San Jose, CA), coordinated the donation of homemade masks for essential workers, and have logged a lot of miles walking and running since the gym is closed. Like others, I've noticed more money in my bank accounts than usual since there is nowhere to shop. This is juxtaposed by an impulse purchase of a Bowflex (Vancouver, WA) elliptical and an exercise bike that spurred the creation of a home gym.

**How Has My Normal Schedule Changed?**

I rarely drive anymore. There are no appointments, lunches, or meetings to attend. There is no travel. There is also no traffic, no lines, and no waiting, for anything. I'm never late for meetings. Today, several of my favorite retail stores shut down their websites because of supply-chain issues. I just learned that The *Aesthetic Surgery Journal* can no longer print and ship journals internationally, because the supply-chain has been interrupted globally.

**What Lessons Have I Learned?**

Time is long. The hours pass slowly in quarantine. Turning on the news to see the nightly reports from New York Governor Andrew Cuomo and New Jersey Governor Phil Murphy reaffirms that staying home is the right thing to do. But it doesn't make this new reality any easier. I am spoiled. As Americans, we are all spoiled. We can have anything we want with a few clicks, sometimes in a matter of hours if not days. But spending all this time at home, without any of those "things" has made me realize how completely unnecessary nearly all of those "things" truly are. Things and stuff are no longer available. I cannot get my nails done or get a haircut or grab lunch with a friend. Yet, I am surviving. We all are surviving. In fact, without all these things and the staff and supply chain resources needed to provide them, right now, we are saving lives. We are doing our part to flatten the curve by staying home and curtailing unnecessary spending that places demands on an already sensitive supply chain. I have also learned how carefully we must protect the very young and the geriatric population plus those who are immunocompromised, during this challenging time. We should look after our elderly neighbors, while maintaining social distance rules, to ensure they have everything they need.

Accepted Manuscript

**How Can We All Roll Up Our Sleeves and Help?**

This is my first time navigating a pandemic. I am social by nature and for extroverts like me, it has been particularly challenging to stay home alone. But it has given me extra time to think about how I can help, what I can contribute to the fight, and to encourage and applaud others doing the same. Some are making masks for healthcare workers. Others are raising money for clothing for doctors and nurses, so they have something clean to change into before they go home to their families. Restaurant owners are feeding out-of-work wait staff by the thousands, all through money donated via GoFundMe (Redwood City, CA). Plastic surgeons are donating ventilators, masks, gowns, and gloves to local hospitals and other crucial supplies, and a field hospital was recently constructed in Central Park to help New York patients. I've found myself attending charity Zoom events and donating to causes that are directing funds to those in need right now, and it feels good to help in this small way. With millions of workers furloughed or unemployed because of the pandemic, a change how we do things is inevitable.

This is a time for innovation. The Aesthetic Society recognized the need to help its members and to ensure consistent and accurate messages were being distributed. They started running a COVID-19 webinar series to help aesthetic surgeons navigate the pandemic and offer critical tools, resources, and advice. The *Aesthetic Surgery Journal* launched *ASJ* Virtual Grand Rounds on Friday, April 3, 2020—an educational webinar featuring leaders in our field presenting on topics in aesthetic surgery. And on April 21 it launched ASJ GEMS (Global Educational Meetings) focusing on learning from and teaching our international colleagues. Both series have been very well attended. Since many doctors have extra time right now, residents, fellows, and other attendees are gaining from the benefit of their wisdom and are engaging with them during the Zoom calls. We are banding together in a way I haven't seen since September 11, 2001. And as if social media does not already play a big part in our lives, we are now relegated to celebrating important events such as birthdays, anniversaries, etc. digitally. Zoom is our new reality. Even grandparents are doing it. Doctors are using Telehealth to offer virtual consults and continuity of care patient care and continue to connect, educate, and engage with them through videos on Instagram, Snapchat, Twitter, and Facebook. We adapt or we die, and at this task of adaptation, plastic surgeons have already begun to excel.

This new reality is a marathon, not a sprint. It will get worse before it gets better. Estimates of when this will end, and life will return to normal are just that—best guesses. We are battling an enemy we cannot see and the information about it seems to change as quickly as we hear it. As of this week, the curve is beginning to flatten with talks about re-opening some states early as May 4, including Vermont, West Virginia, Montana and Hawaii.

Accepted Manuscript

Worldwide as of April 23, 2020, there have been 2,661,504 confirmed cases, 185,504 deaths, and 730,725 who have recovered from COVID-19.[1] The US has had the most cases (849,092,),[2] followed by Spain (213,024) and Italy (187,327). The US has had the most deaths (47,684) followed by Italy (25,085) and Spain (22,157).[1,2]

Dr. Andrew Kornstein said, "How often in our lives are we fortunate enough to identify a person or experience that becomes a prevailing current for the rest of our lives—a soulmate, a financial windfall or disaster, spiritual enlightenment, stardom, working as part of a unit (eg, in the military), or finding a calling, such as medicine?"[3] Years from now, we are likely to reflect and realize this pandemic changed the course of our lives. That is the focus of a new Editorial in the *Aesthetic Surgery Journal* by Dr. Mark Jewell and co-authors who discuss practice management during a pandemic and the issues affecting plastic surgeons.[4]

Thousands of plastic surgeons have been forced to close their practices and furlough their loyal staff without any promise of when they might return. We are in unprecedented times, and we're in it together. Dr. Lorne Rosenfield serves as Section Editor for 'Second Thoughts on First Thoughts' that launched recently in this journal. He wrote an Editor's Note[5] that talks about the learning curve we are experiencing right now and how it is only after the fact can we connect the seminal dots of our past and truly understand what we lived through. We are still learning, but we are also innovating, growing, improving, and hopefully heading toward the peak of COVID-19 and a completely flattened curve.

Stay strong, be brave, and let's all do our part.

Accepted Manuscript

**REFERENCES**

1. Worldometer. COVID-19 Coronavirus Pandemic. Worldometer website. https://www.worldometers.info/coronavirus/. April 2020. Accessed April 6, 2020.
2. Centers for Disease Control. COVID-19 Coronavirus Pandemic. https://www.cdc.gov/coronavirus/2019-ncov/index.html. Accessed April 6, 2020.
3. Kornstein A. My transformation as a trainee in a Halstedian surgical residency: A tribute to my mentors. *Aesthet Surg J Open Forum.* 2019;1(4).
4. Jewell ML, Jewell M, Singer R. Practice Management During a Pandemic: Common Issues that Affect All of Us. *Aesthet Surg J Open Forum.* 2020. In press.
5. Rosenfield L. On Second Thought. *Aesthet Surg J Open Forum.* 2020; 2(1).

Accepted Manuscript
